# Effect of postoperative residual astigmatism on visual outcomes after trifocal intraocular lens implantation

**DOI:** 10.3389/fmed.2023.1202793

**Published:** 2023-07-11

**Authors:** Limei Zhang, Wenqian Shen, Jiying Shen, Min Wang, Shuang Ni, Haike Guo, Jin Yang

**Affiliations:** ^1^Department of Ophthalmology, Shanghai Heping Eye Hospital, Shanghai, China; ^2^Department of Ophthalmology and the Eye Institute, Eye and Ear, Nose, and Throat Hospital, Fudan University, Shanghai, China; ^3^Key National Health Committee Laboratory of Myopia, Fudan University, Chinese Academy of Medical Sciences, Shanghai, China; ^4^Shanghai Key Laboratory of Visual Impairment and Restoration, Shanghai, China

**Keywords:** astigmatism, subjective optometry, uncorrected distance visual acuity, Visual Function Index, Strehl ratio, visual outcomes

## Abstract

**Purpose:**

The aim of this study was to evaluate the effect of residual astigmatism on postoperative visual outcomes after trifocal intraocular lens implantation.

**Methods:**

In this prospective observational study, we divided 156 eyes into two groups according to postoperative astigmatism measured by subjective optometry and followed them up for 3 months. Visual acuity, modulation transfer function (MTF) curves, Strehl ratio (SR), Visual Function Index-14 scores, and photic phenomena were compared.

**Results:**

Linear regression analysis revealed a weak correlation between residual astigmatism and uncorrected distance visual acuity (UDVA) (r = 0.190, *P* = 0.016) at 3 months and a significant between-group difference at 1- and 3-month postoperative UDVA (*P* = 0.038, *P* = 0.018, respectively). MTF curve values and SR (MTF-10 total, MTF-10 cornea, MTF-30 total, MTF-30 cornea, SR Total, and SR cornea) were significantly worse (*P* < 0.001), and the Visual Function Index-14 scores were lower in the 0.5 < astigmatism ≤ 1.25 D group (*P* < 0.05) than in the astigmatism ≤ 0.5 D group. No significant differences were found in the frequency, severity, and bothersomeness of photic phenomena (*P* > 0.05).

**Conclusion:**

Postoperative residual astigmatism affects the UDVA of the trifocal intraocular lens-implanted eyes. Although we found no significant differences in uncorrected intermediate and near visual acuity, both objective and subjective visual quality were affected, suggesting the need for surgical planning when the anticipated postoperative astigmatism is >0.5 D.

## Introduction

The implantation of trifocal intraocular lenses (IOLs) is widely accepted as a reliable new surgical method for patients with presbyopia or cataract (1). Trifocal IOLs provide good whole-course visual acuity (VA) and increase spectacle independence, usually improving the patients' quality of life (2, 3). However, some patients have complained of postoperative visual discomfort after trifocal IOL implantation owing to astigmatism, myopia, hyperopia, and/or higher-order aberrations (4–7). Another study has noted that residual astigmatism occurs in 63% of patients after trifocal IOL implantation (2).

Astigmatism > 1.0 D before cataract surgery is prevalent in almost 50% of cases, and ~90% of patients exhibit postoperative astigmatism ≥ 0.5 D, according to a vector analysis of a large population (8, 9). With the increasing use of multifocal IOLs (MIOLs), several studies have noted the criticality of preoperative corneal astigmatism pertaining to the choice of a trifocal diffractive IOL (10, 11). Generally, patients with preoperative corneal astigmatism > 1.0 D should be corrected with astigmatic keratotomy before MIOL implantation or using a toric-corrected MIOL (11). Despite attempts to eliminate astigmatism during surgical procedures, residual astigmatism after MIOL surgery is inevitable (4, 10). Previous studies have noted that astigmatism can, to a certain degree, improve near vision in eyes with monofocal IOLs, as myopic astigmatism enhances the depth of focus (12, 13); however, there is a lack of research on MIOLs. It has also been reported that residual astigmatism considerably undermines postoperative visual performance and leads to high dissatisfaction rates, regardless of the type of MIOL (5, 10, 14, 15). Most current studies on MIOLs are based on bifocal IOLs, and, to date, few studies have demonstrated the effect of residual astigmatism on visual outcomes after trifocal IOL implantation. Moreover, most earlier studies (3, 10) simulated astigmatism postoperatively by adding cylindrical lenses to evaluate its effect; this approach has, however, certain limitations as the human visual system is not a simple optical instrument, and additional blurring might be induced as the procedure also changes the spherical equivalent.

Therefore, this study aimed to investigate the effect of true residual astigmatism on visual acuity and visual quality with diffractive trifocal IOLs. We also attempted to further explore the extent to which astigmatism is tolerated in patients after trifocal IOL implantation, providing a basis for using trifocal IOLs in clinical practice as an acceptable limitation of astigmatism that should be set preoperatively.

## Materials and methods

### Subject selection and data collection

In this prospective observational study, we included 156 eyes of 156 patients who underwent cataract surgery or refractive lens exchange (RLE), as well as the implantation with the AcrySof IQ PanOptix TFNT00 (Alcon Vision LLC) IOL, from April 2020 to July 2022 at the Shanghai Heping Eye Hospital, China. The inclusion criteria are as follows: (1) age being 32–75 years; (2) implantation of a trifocal IOL; (3) postoperative residual astigmatism within 0–1.25 D and refractive sphere within −0.5 to 0.5 D according to subjective optometry measured at 5 m; and (4) postoperative corrected distant visual acuity ≤ logMAR 0.1. The key exclusion criteria were amblyopia, a history of corneal disease, a history of retinal detachment, a small pupil, neuro-ophthalmic disease, a history of ocular surgery or ocular trauma, and intraoperative or postoperative complications unrelated to the design of the IOL, which may impair visual outcomes through, for example, intraoperative posterior capsule rupture with anterior vitrectomy and cystoid macular edema in some cases. Patients were enrolled under the same conditions and followed up for 3 months. Patients were divided into two groups according to astigmatism measured by subjective optometry at 1 month postoperatively (group A, astigmatism ≤ 0.5 D; group B, 0.5 < astigmatism ≤ 1.25 D). Patients whose residual astigmatism was not within the range of groups A and B were subsequently excluded at 1 week and 3 months postoperatively, respectively. Moreover, at 3 months postoperatively, patients with an astigmatism > 0.5 and ≤ 1.25 D were further divided into subgroups according to axial length (<26 and ≥26 mm) and axis (astigmatism with rule, astigmatism against rule, and oblique astigmatism). Two senior ophthalmologists (L.M.Z. and S.N.) completed all optometry procedures and recorded the data. The study was approved by the Shanghai Heping Eye Hospital and was conducted in adherence to the principles of the Declaration of Helsinki. Informed consent was obtained from all patients before they were included in the study.

### Preoperative examination

Comprehensive preoperative ophthalmologic examinations included visual acuity (VA), intraocular pressure measurement, manifest refraction, slit lamp, corneal tomography (Pentacam HR, OCULUS Optikgerate, Wetzlar, Germany), ocular biometric measurements (IOL-Master 700, Carl Zeiss Meditec, Jena, Germany), optical coherence tomography (Cirrus HD-OCT, Carl Zeiss Meditec, Dublin, CA), B-scan ultrasonography, optomap imaging (Optos Dayton, Carl Zeiss Meditec AG), and fundoscopy.

### Surgical technique

Cataract surgery was performed by an experienced surgeon (J.Y.) using a standardized surgical technique under surface anesthesia. The surgical technique included a 2.2-mm corneal incision in the 10 o'clock direction or the steepest meridian, a capsulorrhexis diameter of ~5.0 mm, hydrodissection, phacoemulsification, irrigation/aspiration of cortical remnants, and an implantation of the IOL in the capsular bag. Femtosecond laser-assisted astigmatic keratotomy procedures were used to correct astigmatism in patients with preoperative corneal astigmatism of 0.75–1.5 D. Postoperative target refraction was set between −0.50 and 0 D according to axial length using the Barrett formula in an IOL power calculation.

### Postoperative follow-up and assessments

Patients were examined at 1 week, 1 month, and 3 months after trifocal IOL implantation. Uncorrected distance visual acuity (UDVA), corrected distance visual acuity (CDVA; measured at 5 m), uncorrected intermediate visual acuity (UIVA; measured at 60 cm), and uncorrected near visual acuity (UNVA; measured at 40 cm) were converted to the logarithm of the minimum angle of resolution (LogMAR). Objective optical quality, including the Strehl ratio (SR) and modulation transfer function (MTF) curve, was evaluated using the HOYA iTrace ray-tracing system (Tracey Technologies, Houston, TX) at a natural pupil size; the data for a 3-mm pupil size were chosen according to the average pupil size of a normal person under natural light. The Quality of Vision (QoV) questionnaire developed by McAlinden et al. (16) was used at the last postoperative visit to evaluate photic phenomena. Patients were asked to respond with not at all (0), a little (1), quite (2), or very (3) to evaluate how they were bothered by the listed symptoms. Patient satisfaction was assessed using the Visual Function Index-14 (VF-14) questionnaire (17) at 3 months postoperatively. The VF-14, which ranges from 0 to 100, determines how troublesome vision-related daily activities (e.g., reading and driving) are for patients, with a higher score representing better functioning.

### Statistical analysis

Measurement data were expressed as means ± SD. The Kolmogorov-Smirnov test was used to assess normality, and independent-sample *t*-tests and one-way analysis of variance were used to compare differences in visual acuity and visual quality, both preoperatively and postoperatively, between groups. When the variables were not normally distributed, the nonparametric Mann-Whitney U test was used instead. Relationships between variables were analyzed with the bivariate correlation models and Spearman's correlation coefficients. Group sample sizes of 95 and 61 achieve 74.815% power to reject the null hypothesis of equal means when the population's mean UDVA difference is 0.022 with a standard deviation for both groups of 0.050 and with a significance level (alpha) of 0.050 using a two-sided two-sample equal-variance *t*-test. In all tests, statistical significance was assumed at a threshold of a *P*-value of <0.05. All statistical analyses were performed using SPSS version 26 (IBM, New York, USA).

## Results

### Baseline characteristics

Of the 182 enrolled patients, 26 were excluded for the following reasons: refusal to participate (*n* = 4), incomplete clinical information or loss of follow-up (*n* = 17), and surgical complications (*n* = 5). Thus, 156 eyes of 156 patients were included in this study, with 95 eyes in group A (astigmatism ≤ 0.5 D) and 61 in group B (0.5 < astigmatism ≤ 1.25 D). Patients were followed up for 3 months postoperatively. Table 1 shows the demographic and preoperative characteristics of both groups. The mean age of the patients was 56.70 ± 8.20 years (group A) and 58.81 ± 9.29 years (group B). No significant differences in age, preoperative axial length, anterior chamber depth, lens thickness, white-to-white ratio, or IOL power values (all *P* > 0.05) were noted between both groups. There were also no significant between-group differences in preoperative UDVA or CDVA (all *P* > 0.05).

**Table 1:** Baseline characteristics of patients in the study.

**Characteristics, mean ±SD**	**Astigmatism ≤ 0.5 D (group A, *n* = 95)**	**0.5 < astigmatism ≤ 1.25 D (group B, *n* = 61)**	***P*-value**
Age, years	56.70 ± 8.20	58.81 ± 9.29	0.143
Men/women, *n*	43/52	33/28	
Left/right, *n*	45/50	34/27	
Axial length, mm	25.31 ± 2.06	24.89 ± 1.72	0.157
Anterior chamber depth, mm	3.28 ± 0.40	3.20 ± 0.34	0.154
Lens thickness, mm	4.33 ± 0.43	4.40 ± 0.34	0.246
White-to-white, mm	11.92 ± 0.43	11.87 ± 0.46	0.482
IOL power, diopter	16.54 ± 5.47	17.71 ± 4.80	0.156
UDVA, logMAR	0.80 ± 0.46	0.76 ± 0.48	0.572
CDVA, logMAR	0.28 ± 0.22	0.28 ± 0.23	0.792

SD, standard deviation; IOL, intraocular lens; UDVA, uncorrected distance visual acuity; CDVA, corrected distance visual acuity; logMAR, log of the minimum angle of resolution.

### Visual outcomes

**Figure 1:**
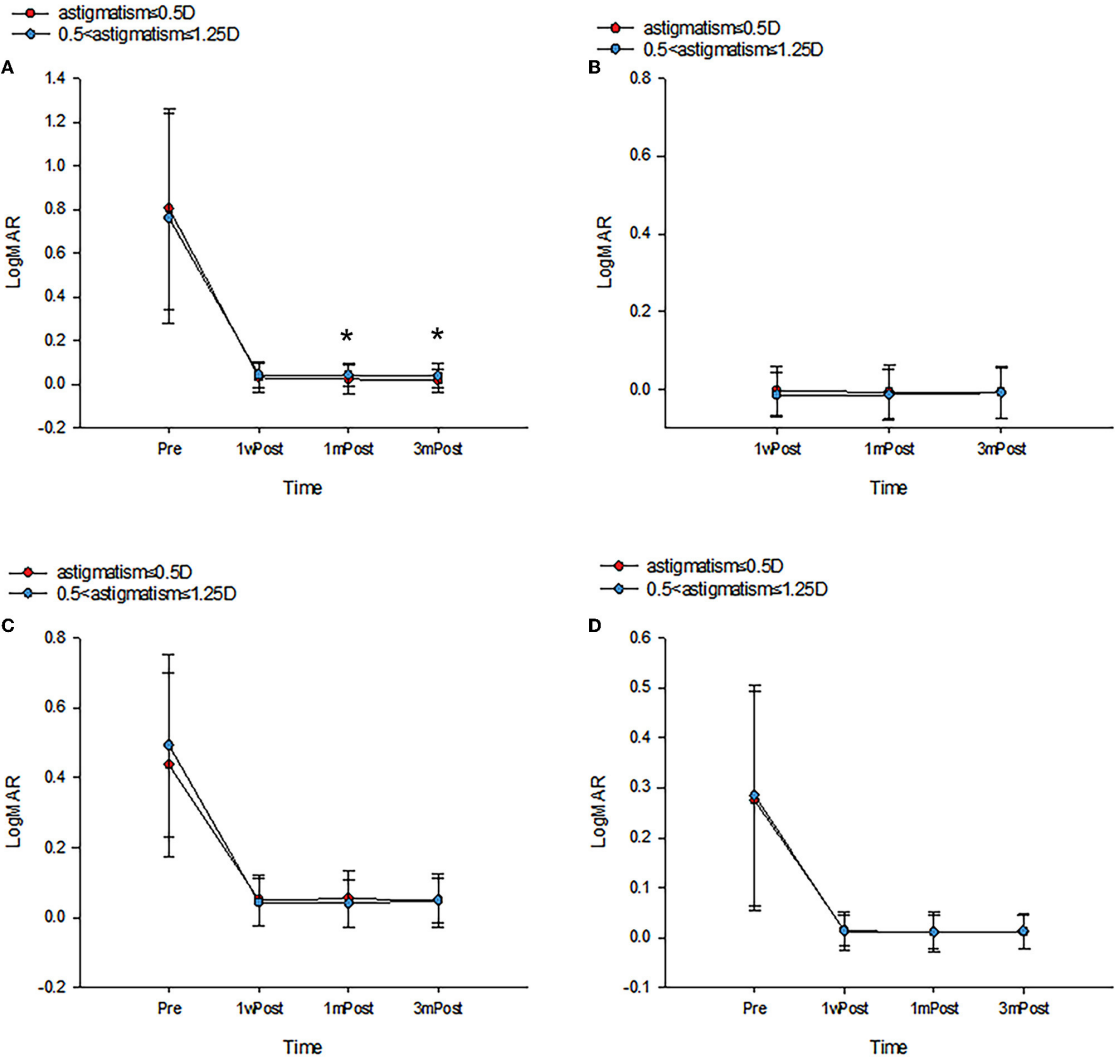
The preoperative and postoperative visual outcomes of two groups at 1 week, 1 month, and 3 months. All data were presented as mean ± SD. **(A)** UDVA (logMAR), **(B)** UIVA (logMAR), **(C)** UNVA (logMAR), and **(D)** CDVA (logMAR). *P < 0.05.

**Figure 2:**
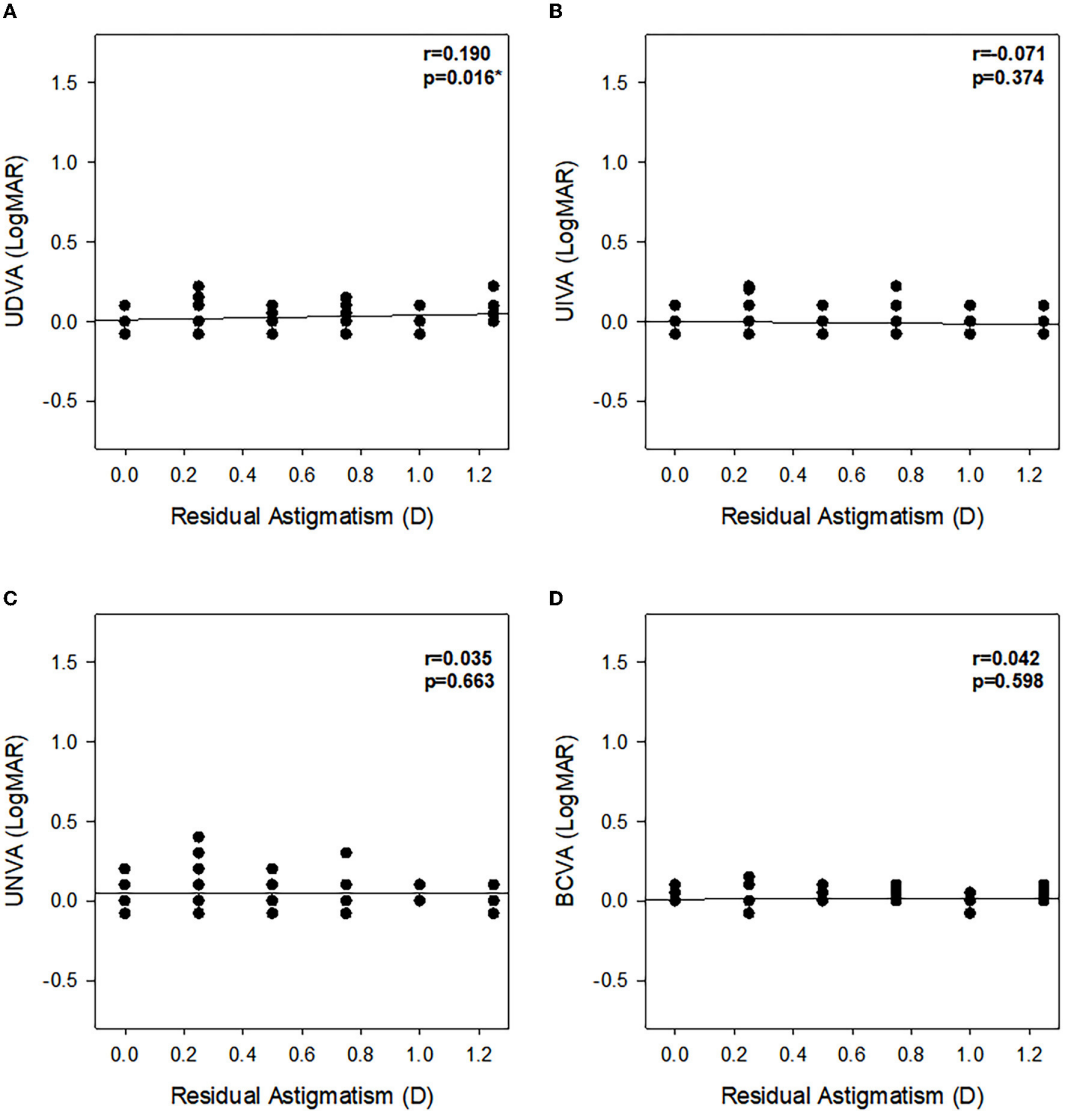
Assessment of the relationship between residual astigmatism and VA (LogMAR) at 3 months postoperatively. **(A)** UDVA (logMAR), **(B)** UIVA (logMAR), **(C)** UNVA (logMAR), and **(D)** CDVA (logMAR). r, Pearson's correlation coefficient; *P < 0.05.

Figure 1 summarizes the mean UDVA, UIVA, UNVA, and CDVA of the two groups at different postoperative time points. The refractive status of both groups was near the target diopter with high refractive predictability. All values significantly improved postoperatively. There was a significant difference at 1- and 3-month postoperative UDVA (0.025 ± 0.064 vs. 0.044 ± 0.055, *P* = 0.038, 0.018 ± 0.055 vs. 0.039 ± 0.057, *P* = 0.018, respectively) and defocus equivalent (−0.108 ± 0.296 vs. −0.355 ± 0.278, *P* < 0.001, −0.103 ± 0.283 vs. −0.344 ± 0.291, *P* < 0.001, respectively) between the two groups; however, UIVA and UNVA were not significantly affected by uncorrected astigmatism at all the time points (all *P* > 0.05). Figure 2A displays the linear regression analysis revealing a weak correlation between 3-month postoperative residual astigmatism and UDVA (r^2^ = 0.190, *P* = 0.016). No significant correlations were observed between postoperative residual astigmatism and UIVA (Figure 2B, r^2^ = −0.071, *P* = 0.374), UNVA (Figure 2C, r^2^ = 0.035, *P* = 0.663), or CDVA (Figure 2D, r^2^ = 0.042, *P* = 0.598).

### Objective visual quality

The MTF-10 total (0.468 ± 0.126 vs. 0.288 ± 0.107), MTF-10 cornea (0.608 ± 0.180 vs. 0.464 ± 0.158), MTF-30 total (0.134 ± 0.070 vs. 0.088 ± 0.032), MTF-30 cornea (0.198 ± 0.117 vs. 0.129 ± 0.087), SR total (0.204 ± 0.110 vs. 0.106 ± 0.045), and SR cornea (0.301 ± 0.199 vs. 0.197 ± 0.142) were higher in group A than in group B, and the differences were significant (all *P* < 0.001). However, no significant differences were noted in SR internal, MTF-10 internal, or MTF-30 internal between both groups (all *P* > 0.05) (Table 2). Patients with astigmatism > 0.5 D and ≤ 1.25 D were further divided into two groups according to the axial length (<26 and ≥26 mm) (Table 3). A significant difference was found in MTF-30 total (0.095 ± 0.031 vs. 0.074 ± 0.028, *P* < 0.05) but not in MTF-10 total, MTF-10 cornea, MTF-30 cornea, SR total, or SR cornea (all *P* > 0.05). Patients with astigmatism > 0.5 D and ≤ 1.25 D were subdivided into those with with-the-rule astigmatism, those with against-the-rule astigmatism, and those with oblique astigmatism to investigate the effect of the axis (Table 4), although no significant differences between the three subgroups were found in MTF (MTF-10 total, MTF-10 cornea, MTF-10 internal, MTF-30 total, MTF-30 cornea, and MTF-30 internal) or SR (SR total, SR cornea, and SR internal) (all *P* > 0.05).

**Table 2:** MTF-10, MTF-30, and SR of the two groups in the 156 eyes of 156 patients at 3 months postoperatively.

**Parameter, mean ±SD**	**Astigmatism ≤ 0.5 D (group A, *n* = 95)**	**0.5 < astigmatism ≤ 1.25 D (group B, *n* = 61)**	***P*-value**
MTF-10 total	0.468 ± 0.126	0.288 ± 0.107	< 0.001^**^
MTF-10 cornea	0.608 ± 0.180	0.464 ± 0.158	< 0.001^**^
MTF-10 internal	0.480 ± 0.150	0.425 ± 0.168	0.082
MTF-30 total	0.134 ± 0.070	0.088 ± 0.032	< 0.001^**^
MTF-30 cornea	0.198 ± 0.117	0.129 ± 0.087	< 0.001^**^
MTF-30 internal	0.142 ± 0.064	0.133 ± 0.071	0.527
SR total	0.204 ± 0.110	0.106 ± 0.045	< 0.001^**^
SR cornea	0.301 ± 0.199	0.197 ± 0.142	< 0.001^**^
SR internal	0.220 ± 0.107	0.206 ± 0.129	0.572

MTF, modulation transfer function; SR, Strehl ratio;

** *P* < 0.001.

**Table 3:** MTF-10, MTF-30, and SR of patients with different axial lengths at 3 months postoperatively (0.5 < astigmatism ≤ 1.25 D).

**Parameter, mean ±SD**	**0.5 < astigmatism ≤ 1.25 D, AL < 26 mm (*n* = 41)**	**0.5 < astigmatism ≤ 1.25 D, AL ≥26 mm (*n* = 20)**	***P*-value**
MTF-10 total	0.308 ± 0.093	0.254 ± 0.125	0.115
MTF-10 cornea	0.438 ± 0.156	0.511 ± 0.155	0.112
MTF-10 internal	0.456 ± 0.151	0.370 ± 0.188	0.096
MTF-30 total	0.095 ± 0.031	0.074 ± 0.028	0.019^*^
MTF-30 cornea	0.111 ± 0.061	0.161 ± 0.116	0.090
MTF-30 internal	0.144 ± 0.072	0.114 ± 0.065	0.135
SR total	0.115 ± 0.041	0.095 ± 0.050	0.069
SR cornea	0.174 ± 0.121	0.239 ± 0.169	0.146
SR internal	0.218 ± 0.132	0.184 ± 0.124	0.350

MTF, modulation transfer function; SR, Strehl ratio; AL, axial length;

* *P* < 0.05.

**Table 4:** MTF-10, MTF-30, and SR of patients with a different axis of astigmatism at 3 months postoperatively (0.5 < astigmatism ≤ 1.25 D).

**Parameter, mean ±SD**	**WTR (*n* = 20)**	**OBL (*n* = 23)**	**ATR (*n* = 18)**	***P*-value**
MTF-10 total	0.268 ± 0.109	0.289 ± 0.113	0.298 ± 0.101	0.739
MTF-10 cornea	0.520 ± 0.197	0.490 ± 0.118	0.404 ± 0.164	0.103
MTF-10 internal	0.379 ± 0.146	0.424 ± 0.161	0.452 ± 0.188	0.467
MTF-30 total	0.081 ± 0.032	0.086 ± 0.032	0.095 ± 0.031	0.480
MTF-30 cornea	0.164 ± 0.090	0.140 ± 0.102	0.098 ± 0.068	0.108
MTF-30 internal	0.100 ± 0.033	0.131 ± 0.065	0.150 ± 0.083	0.100
SR total	0.098 ± 0.047	0.100 ± 0.033	0.115 ± 0.047	0.538
SR cornea	0.258 ± 0.156	0.216 ± 0.164	0.144 ± 0.098	0.083
SR internal	0.157 ± 0.083	0.201 ± 0.121	0.234 ± 0.148	0.214

MTF, modulation transfer function; SR, Strehl ratio; WTR, with the rule; OBL, oblique astigmatism; ATR, against the rule.

### Quality of life and photic phenomena

**Figure 3:**
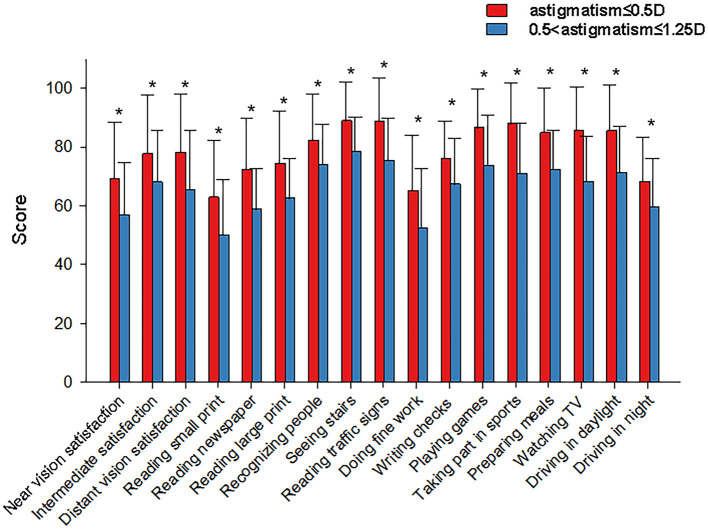
Subjective visual quality questionnaire classification of the bar chart of the two groups at 3 months postoperatively. X-axis, VF-14 questionnaire; Y-axis, score of visual satisfaction; and *P < 0.05.

**Figure 4:**
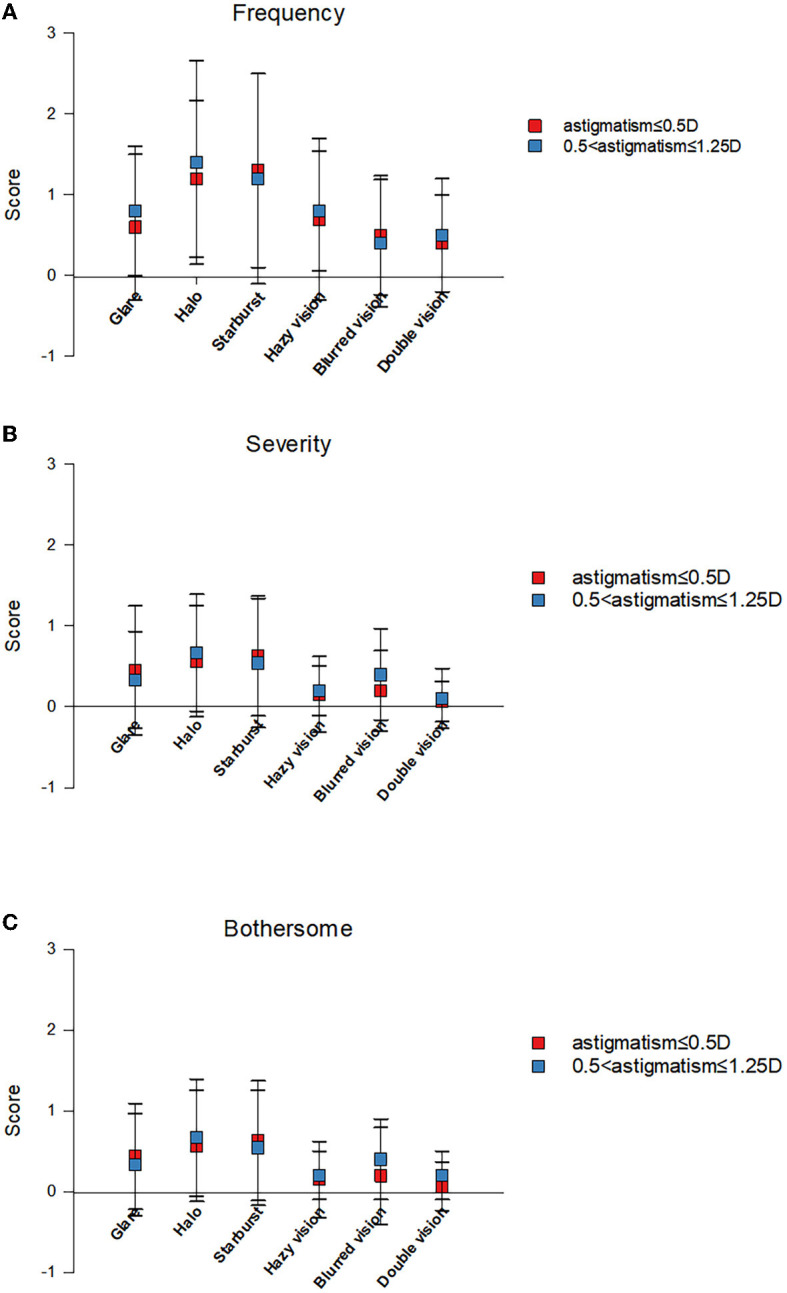
Quality of vision scores obtained with the questionnaire by McAlinden et al. for frequency **(A)**, severity **(B)**, and bothersomeness **(C)** of different visual symptoms.

The VF-14 questionnaire was used to assess subjective visual quality at 3 months postoperatively (Figure 3). The mean VF-14 score was significantly higher in group A than in group B (78.44 ± 16.32 vs. 66.16 ± 16.09, *P* < 0.05). The mean patient satisfaction scores for distance, intermediate, and near visual acuity were 78.04 vs. 65.28, 77.70 vs. 68.06, and 69.26 vs. 56.94, respectively (all *P* < 0.05). Compared with group B, group A scored significantly higher on quality-of-life items (*P* < 0.05). Compared with satisfaction pertaining to all items, satisfaction with reading small prints and doing fine handwork was relatively low (Figure 3). Figure 4 shows findings on photic phenomena for both groups 3 months after trifocal IOL implantation. Patients were required to identify the frequency and severity of photic occurrences, as well as how bothersome they were in their daily life. The frequency, severity, and bothersomeness of dysphenopsia, including glare, halo, starburst, hazy vision, blurred vision, and double vision, did not significantly differ between both groups (all *P* > 0.05). Similarly, no significant difference was found in the presence of photic phenomena between both groups (*P* > 0.05).

### Complications

Transient intraocular pressure was reported in one eye (0.6%) in group A. Other postoperative surgical complications included cystoid macular edema in two eyes (1.2%), a posterior capsular rupture in one eye (0.6%), and poor neuroadaptation in one eye (0.6%) in group B. These eyes were not included in the statistical analyses.

## Discussion

This study was conducted to evaluate the effect of postoperative residual astigmatism on visual outcomes and to investigate the allowable limit of astigmatism to achieve sufficient visual acuity in the eyes after trifocal IOL implantation. We found a significant difference in UDVA, objective visual quality, and patient satisfaction between patients with residual astigmatism of ≤ 0.50 D and those with astigmatism of >0.50 D. Our findings confirm the independent role of astigmatism in the visual outcomes of pseudophakic patients, even at low astigmatism levels. To the best of our knowledge, this study is the first to clarify the effect of true postoperative residual astigmatism on the subjective and objective visual quality of patients with trifocal IOLs.

In our study, UDVA was higher in group A (astigmatism ≤ 0.5 D) than in group B (0.5 < astigmatism ≤ 1.25 D) at 1 and 3 months postoperatively, suggesting that the distant vision in the eyes implanted with trifocal IOL was significantly affected by residual astigmatism, which is consistent with the findings of previous studies (14, 18). No statistical difference was identified between the two groups in UDVA at 1 week postoperatively, which might be explained by the early unstable postoperative corneal morphology or other factors. As Hayashi et al. (3) demonstrated, when postoperative residual astigmatism increased to 1.5 D, not only UDVA but also UIVA and UNVA correspondingly deteriorated in trifocal IOL-implanted eyes. However, in our current study, residual astigmatism was only weakly correlated with postoperative UIVA and UNVA. This discrepancy might be associated with the fact that we studied patients with astigmatism of ≤ 1.25 D.

As vision acuity reflects the effects of macular cones and the brain's nervous system, the measurements of vision are considerably limited and subjective (19, 20). Thus, to explore the potential effect of astigmatism on optical quality, we quantified objective visual quality using MTF curves, SR, and other indicators. MTF reflects the sharpness of images at different spatial frequencies. A low spatial frequency usually reflects the ability to view the object's contour, whereas a high spatial frequency reflects the ability to distinguish fine objects (21). Differences in objective visual quality (SR total, SR cornea, MTF-10 total, MTF-10 cornea, MTF-30 total, and MTF-30 cornea) were observed between the two groups at 3 months postoperatively, indicating that the visual quality of patients was affected by an increase in postoperative residual astigmatism. As residual astigmatism increased, the patients' ability to distinguish the contours and details of objects decreased (22). Further analysis of the causes affecting visual quality showed statistical differences in the corneal MTF-10, MTF-30, and SR between both groups, indicating that residual corneal astigmatism was the main factor affecting visual quality. Of note, a significantly better MTF-30 total was detected in patients with normal axial length than in those with long axial length, implying a better visual quality at a high spatial frequency and a higher capability to distinguish details to a certain degree of astigmatism in the former. This may be attributed to the density of retinal cells in the posterior pole in high-myopic patients, as this is much lower than that in emmetropic patients. Aside from the magnitude of astigmatism, we analyzed the effect of the astigmatism axis by categorizing patients (0.5 < astigmatism ≤ 1.25 D) into three groups (with the rule, against the rule, and oblique astigmatism) and found that visual quality was not materially impacted by the astigmatism axis, which is in line with the findings of most previous studies (23). However, several earlier studies have noted the dependence of visual acuity on the astigmatism axis (18, 24). We assume that this discrepancy in results stems from differences in sample sizes and different magnitudes of astigmatism. Thus, both our hypotheses regarding the effects of axis and axial length require further research involving larger samples and longer follow-up periods, as the sample size of this current study was relatively small.

Patient satisfaction scores assessed with the VF-14 questionnaire were consistent with the objective visual quality findings. Although both groups achieved relatively good UDVA, UIVA, and UNVA, the visual satisfaction score in group A, which comprised patients with relatively low astigmatism, was significantly higher than that in group B, regardless of the distance, intermediate, or near visual acuity satisfaction. Our results suggest that visual functions, including night driving, reading, reading small prints, and threading a needle, may be affected by astigmatism, even when conventional VA is good. Watanabe et al. (25) reported that astigmatism may be associated with the deterioration of visual functions, even when a conventional VA of 20/20 was attained. Another commonly reported issue with trifocal IOLs is the presence of photic phenomena. Glares, halos, and starbursts are consistently reported as the most frequent and bothersome QoV symptoms after IOL surgery (26, 27). Moreover, previous studies have suggested a higher prevalence of photic phenomena and more significant and frequent occurrences in patients with relatively greater astigmatism (4, 6, 28). In our study, there were no statistical differences between the two groups in the subjective perception of dysphotopsia, including frequency, severity, and discomfort with visual symptoms, which might be attributable to the fact that the questionnaire that we used was only presented to patients at 3 months postoperatively. As some previous studies have reported, the perception of photic phenomena weakens over time, likely due to neuroadaptation processes (29, 30). Moreover, the residual astigmatism we studied was within 1.25 D, predominantly due to the strict control of astigmatism before trifocal IOL implantation, and further research on the effect of higher levels of residual astigmatism (>1.25 D) on dysphotopsia is warranted.

This study has certain limitations. First, as the objects of this study are patients with mild or moderate astigmatism, the residual astigmatism was relatively low, and the difference between the two groups was minor, resulting in the lack of statistical differences in visual acuity between the two groups besides 1 and 3 months postoperative UDVA. Second, we were not able to conduct a comprehensive exploration of the population with postoperative residual astigmatism exceeding 1.25 D. Third, the follow-up was not long enough. Further multicenter studies and longer follow-up periods are required to explore the applicability or generalizability of our results to patients implanted with trifocal IOLs.

## Conclusion

Patients with presbyopia or cataracts achieved stable and good vision after trifocal IOL (AcrySof IQ PanOptix TFNT00 (Alcon Vision LLC)) implantation in our study, but the control of postoperative residual astigmatism is very strict. When the residual astigmatism exceeded 0.5 D, even though intermediate and near visual acuity were not significantly impaired, a remarkable deterioration in objective and subjective visual quality was observed, and the magnitude of astigmatism played the most important role in postoperative visual quality, indicating that postoperative residual astigmatism needs to be controlled within 0.5 D to acquire the desired subjective and objective visual quality.

## Data availability statement

The original contributions presented in the study are included in the article/Supplementary material, further inquiries can be directed to the corresponding authors.

## Ethics statement

The studies involving human participants were reviewed and approved by the Ethics Committee of Shanghai Heping Eye Hospital (protocol code HXYK-SHHP-2020-0004, accessed on March 2, 2020). The patients/participants provided their written informed consent to participate in this study.

## Author contributions

JY and HG designed the research and critically revised the manuscript. LZ, JS, and SN were involved in patient examination and data collection. MW and LZ analyzed the data. LZ and WS wrote the first draft of the manuscript. All authors commented on previous versions of the manuscript. All authors have read and agreed to the published version of the manuscript.
