# Supplementary material for: Effect of postoperative residual astigmatism on visual outcomes after trifocal intraocular lens implantation
Source: Front Med (Lausanne). 2023 Jul 11;10:1202793. doi: 10.3389/fmed.2023.1202793 (PMC10368131; doi:10.3389/fmed.2023.1202793)
Supplement: Supplementary file 1 [file Data_Sheet_1.docx]

Supplementary Material

Effect of Postoperative Residual Astigmatism on Visual Outcomes After Trifocal Intraocular Lens Implantation

Limei Zhang^1†^ , Wenqian Shen^2,3,4†^ , Jiying Shen^1†^ , Min Wang^1^ , Shuang Ni^1^ , Haike Guo^1*^ , and Jin Yang^2,3,4*^

*** Correspondence:**

Jin Yang: [jin_er76@hotmail.com](mailto:jin_er76@hotmail.com)

Haike Guo: [guohaike@hotmail.com](mailto:guohaike@hotmail.com)

## Supplementary Figures


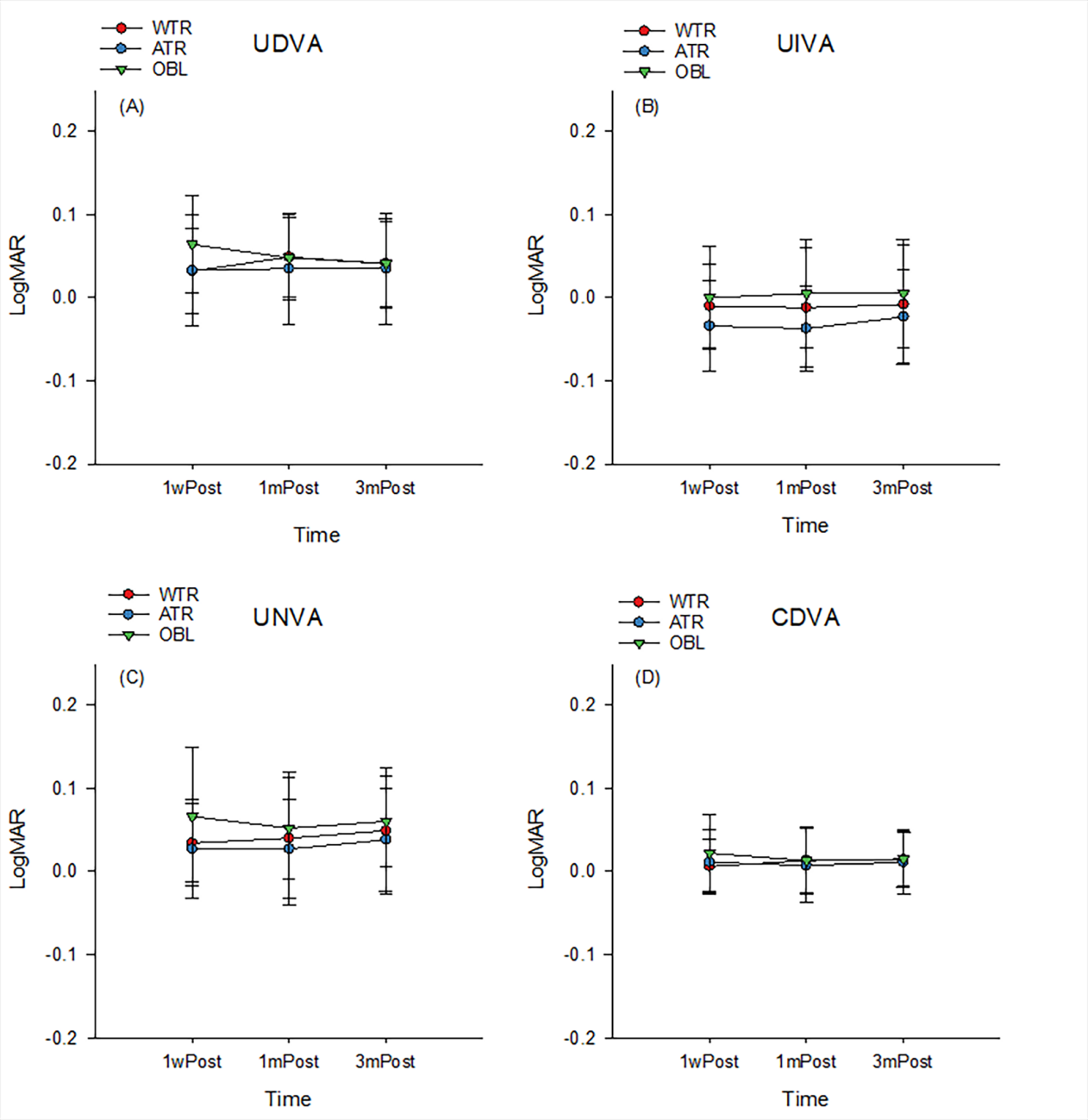


**Supplementary Figure 1.** Comparison of the mean visual acuity of patients with a different axis of astigmatism (0.5<astigmatism≤1.25 D) postoperatively. All data were presented as means ± SDs. (A) UDVA (logMAR). (B) UIVA (logMAR). (C) UNVA (logMAR). (D) CDVA (logMAR). Error bars represent 90% CIs.


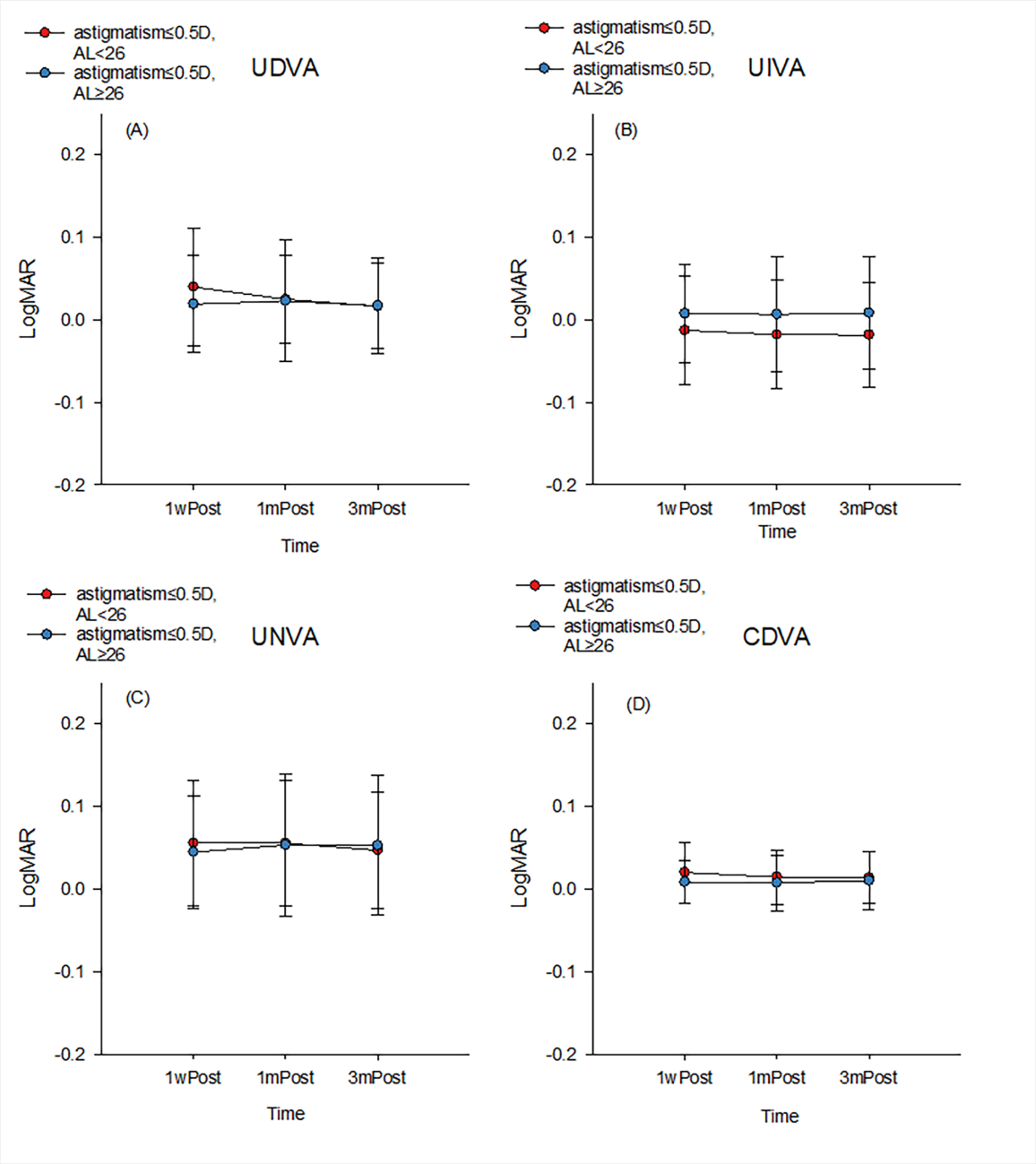


**Supplementary Figure 2.** Comparison of the mean visual acuity of patients with different axial length (astigmatism≤0.5 D) postoperatively. All data were presented as means ± SDs. (A) UDVA (logMAR). (B) UIVA (logMAR). (C) UNVA (logMAR). (D) CDVA (logMAR). Error bars represent 90% CIs.


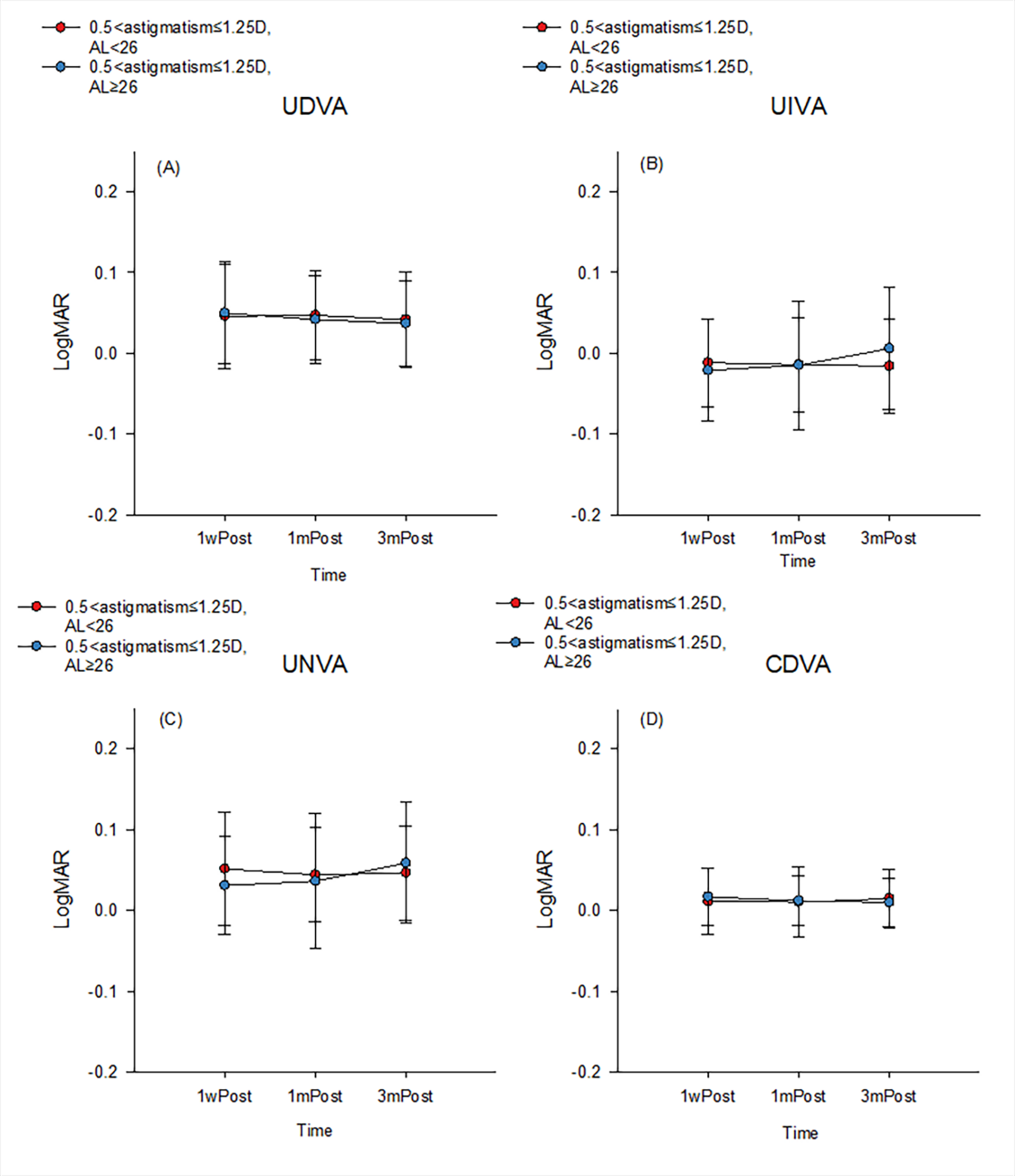


**Supplementary Figure 3.** Comparison of the mean visual acuity of patients with different axial length (0.5<astigmatism≤1.25 D) postoperatively. All data were presented as means ± SDs. (A) UDVA (logMAR). (B) UIVA (logMAR). (C) UNVA (logMAR). (D) CDVA (logMAR). Error bars represent 90% CIs.

**Supplementary Table 1.** Objective outcome comparisons between groups with different magnitudes of residual astigmatism at the postoperative assessment (1 week, 1 month and 3 months).

| **Parameter,**  **mean±SD** | **Astigmatism≤0.5 D**  **(group A, n=95)** | **0.5<Astigmatism≤1.25 D**  **(group B, n=61)** | **P-value** |
| --- | --- | --- | --- |
| 1 week |  |  |  |
| UDVA, LogMAR | 0.031±0.069 | 0.044±0.060 | 0.211 |
| UIVA, LogMAR | -0.003±0.064 | -0.014±0.057 | 0.291 |
| UNVA, LogMAR | 0.051±0.072 | 0.044±0.067 | 0.544 |
| CDVA, LogMAR | 0.014±0.032 | 0.013±0.040 | 0.834 |
| Sphere (D) | 0.047±0.262 | 0.135±0.289 | 0.051 |
| Defocus equivalent (D) | -0.115±0.274 | -0.314±0.302 | 0.000** |
| 1 month |  |  |  |
| UDVA, LogMAR | 0.025±0.064 | 0.044±0.055 | 0.038* |
| UIVA, LogMAR | -0.006±0.068 | -0.013±0.065 | 0.520 |
| UNVA, LogMAR | 0.055±0.081 | 0.041±0.067 | 0.225 |
| CDVA, LogMAR | 0.011±0.033 | 0.011±0.040 | 0.999 |
| Sphere (D) | 0.045±0.279 | 0.107±0.268 | 0.155 |
| Defocus equivalent (D) | -0.108±0.296 | -0.355±0.278 | 0.000** |
| 3 months |  |  |  |
| UDVA, LogMAR | 0.018±0.055 | 0.039±0.057 | 0.018* |
| UIVA, LogMAR | -0.006±0.067 | -0.008±0.065 | 0.840 |
| UNVA, LogMAR | 0.050±0.077 | 0.050±0.064 | 0.933 |
| CDVA, LogMAR | 0.012±0.033 | 0.013±0.034 | 0.781 |
| Sphere (D) | 0.040±0.276 | 0.115±0.283 | 0.096 |
| Defocus equivalent (D) | -0.103±0.283 | -0.344±0.291 | 0.000** |

SD, standard deviation; UDVA, uncorrected distance visual acuity; logMAR, log of the minimum angle of resolution; UIVA, uncorrected intermediate visual acuity; UNVA, uncorrected near visual acuity; CDVA, corrected distance visual acuity; and *P<0.05, **P<0.001
